# 3-Ethyl­sulfanyl-5-methyl-1-phenyl-7-(pyrrolidin-1-yl)-1*H*-pyrimido[4,5-*e*][1,3,4]thia­diazine

**DOI:** 10.1107/S1600536808021144

**Published:** 2008-07-16

**Authors:** M. Nikpour, M. Bakavoli, M. Rahimizadeh, M. R. Bigdeli, M. Mirzaei

**Affiliations:** a Department of Chemistry, School of Sciences, Islamic Azad University, Ahvaz Branch, Ahvaz, Iran; b Department of Chemistry, School of Sciences, Ferdowsi University, Mashhad, 917751436, Iran

## Abstract

In the crystal structure of the title compound, C_18_H_21_N_5_S_2_, the thia­diazine six-membered ring and pyrrolidine five-membered ring display boat and envelope conformations, respectively. The crystal structure contains weak C—H⋯N and C—H⋯S hydrogen bonding.

## Related literature

For general background, see: Rahimizadeh *et al.* (1997 ▶); Elliott (1981 ▶); Bakavoli *et al.* (2006 ▶, 2007 ▶, 2008 ▶).
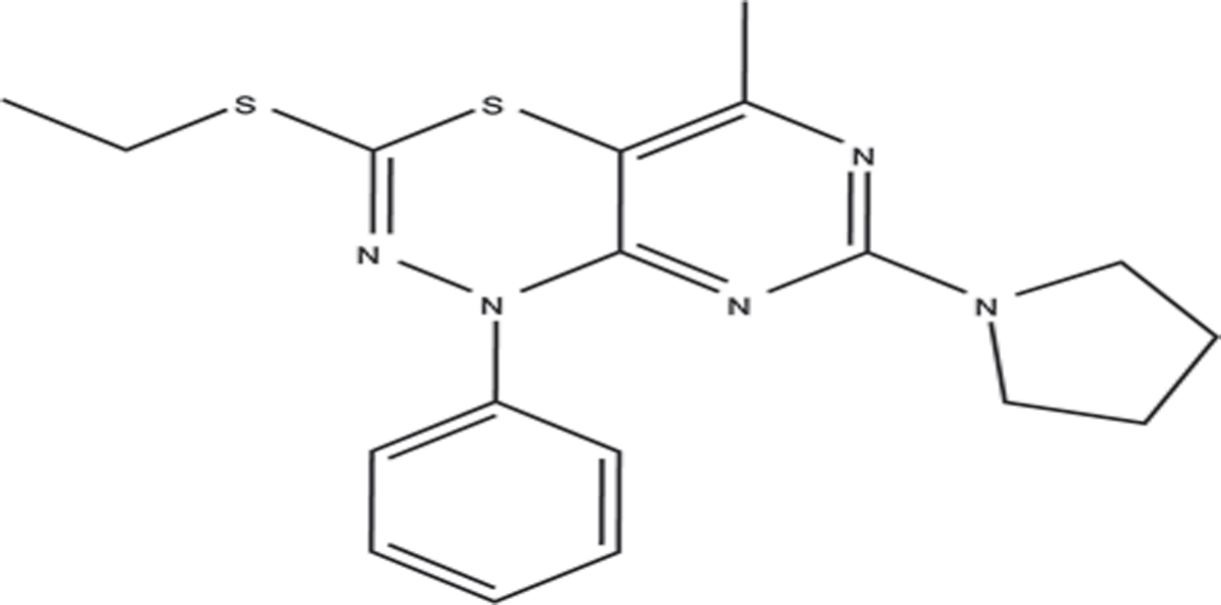


## Experimental

### 

#### Crystal data

C_18_H_21_N_5_S_2_
*M* *_r_* = 371.52
Orthorhombic, 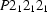

*a* = 8.3601 (2) Å
*b* = 10.3596 (3) Å
*c* = 20.5754 (6) Å
*V* = 1781.98 (8) Å^3^
*Z* = 4
Mo *K*α radiation
μ = 0.31 mm^−1^
*T* = 100 (2) K
0.43 × 0.34 × 0.25 mm

#### Data collection

Bruker APEXII CCD area-detector diffractometer
Absorption correction: multi-scan (*APEX2*; Bruker, 2005 ▶) *T*_min_ = 0.878, *T*_max_ = 0.926
36558 measured reflections
6479 independent reflections
5952 reflections with *I* > 2σ(*I*)
*R*_int_ = 0.042

#### Refinement

*R*[*F* ^2^ > 2σ(*F* ^2^)] = 0.031
*wR*(*F* ^2^) = 0.076
*S* = 1.01
6479 reflections
228 parameters
H-atom parameters constrained
Δρ_max_ = 0.39 e Å^−3^
Δρ_min_ = −0.24 e Å^−3^
Absolute structure: Flack (1983 ▶), 2828 Friedel pairs
Flack parameter: −0.01 (4)

### 

Data collection: *APEX2* (Bruker, 2005 ▶); cell refinement: *APEX2*; data reduction: *APEX2*; program(s) used to solve structure: *SHELXTL* (Sheldrick, 2008 ▶); program(s) used to refine structure: *SHELXTL*; molecular graphics: *SHELXTL*; software used to prepare material for publication: *SHELXTL*.

## Supplementary Material

Crystal structure: contains datablocks I, global. DOI: 10.1107/S1600536808021144/xu2431sup1.cif

Structure factors: contains datablocks I. DOI: 10.1107/S1600536808021144/xu2431Isup2.hkl

Additional supplementary materials:  crystallographic information; 3D view; checkCIF report

## Figures and Tables

**Table 1:** Hydrogen-bond geometry (Å, °)

*D*—H⋯*A*	*D*—H	H⋯*A*	*D*⋯*A*	*D*—H⋯*A*
C12—H12*A*⋯N4^i^	0.95	2.62	3.5630 (15)	172
C15—H15*B*⋯S2^ii^	0.99	2.83	3.6264 (13)	138

Symmetry codes: (i) 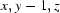; (ii) 
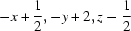
.

**Fig. 1.**
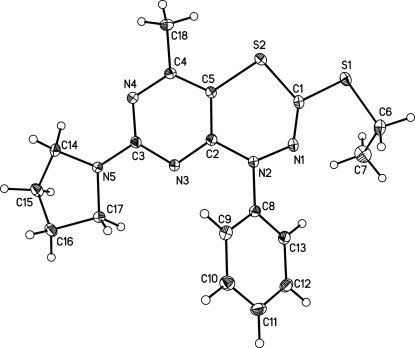
The molecular structure of the title compound with 30% probability displacement (arbitrary spheres for H atoms).

## supplementary crystallographic information

### Comment

The diverse biological activities of pyrimido [4,5-*e*][1,3,4]thiadiazine persuaded us to search for newer and more efficient synthetic methods for this class of heterocyclic compounds. These compounds have been described as being nucleoside analogues, anti-inflammatory, hypotensive, diuretic, and phosphodiesterase inhibitor agents. Despite their importance from pharmacological and synthetic point of views, comparatively few methods for their preparation have been reported. Pyrimido [4,5-*e*] [1,3,4]thiadiazines have been solely synthesized from pyrimidines. Previous routes to such systems have involved condensation of 2,4- dichloro–5-nitro -6-methylpyrimidine with dithizone (Rahimizadeh *et al.*,  1997) *via* Smiles Rearrangement, heterocyclization of 6-hydrazino substituted uracils with isothiocyanates and *N*-bromosuccinimide, reaction of thiohydrazides with 4,5- dihalopyrimidines (Elliott, 1981), condensation of 5-bromo-2-chloro-6-methyl-4-(1-methylhydrazino) pyrimidine with carbondisulfide and alkylhalides (Bakavoli *et al.*,  2007) and isothiocyanates (Bakavoli *et al.*,  2008). In aprevious communication (Bakavoli *et al.*,  2006), we described a new approach for the formation of 1-phenyl-1*H*-[1,3,4]thiadiazino[5,6-*b*]quinoxalines. The synthesis we developed involved heterocyclization of alkyl-2-phenylhydrazinecarbodithioates as bifunctional nucleophiles with 2,3-dichloroquinoxaline as an electrophile. To extend the scope of this strategy, we explored other electrophilic species that could successfully undergo similar reaction.

The molecular structure is shown in Fig. 1. In the title crystal structure, the thiadiazine six-membered ring and pyrrolidine five-membered ring display the boat and envelope configuration, respectively. The crystal structure contains weak C—H···N and C—H···S hydrogen bonding (Table 1).

### Experimental

A mixture of 5-bromo2,4-dichloro-6-methylpyrimidine (2.5 mmol, 0.61 g), each alkyl-2-phenylhydrazinecarbodithioates (2.5 mmol) and triethylamine (1 ml) in acetonitril (10 ml) were boiled under inert atmosphere for 3 h. After the reaction was completed, the mixture was cooled to room temperature, and then evaporated under reduced pressure. The residue was washed with water and crystallized with ethanol and then washed with petroleum ether 40–60 to give pyrimido [4,5-*e*][1,3,4] thiadiazines. A mixture of previous obtained compound (5 mmol) in ethanol (20 ml) was heated under reflux with pyrrolidine (1.8 g) for 4 h. The solvent was removed and the residue was washed with water and then crystallized from ethanol to give the title crystals.

### Refinement

Methyl H atoms were placed in calculated positions with C—H = 0.98 Å and torsion angles were refined to fif the electron density, U_iso_(H) = 1.5U_eq_(C). Other H atoms were placed in calculated positions with C—H = 0.95 (aromatic) and 0.99 Å (methylene), and refined in riding mode with U_iso_(H) = 1.2U_eq_(C).

### Crystal data

**Table:**

C_18_H_21_N_5_S_2_	*D*_x_ = 1.385 Mg m^−^^3^
*M**_r_* = 371.52	Melting point: 407 K
Orthorhombic, *P*2_1_2_1_2_1_	Mo *K*α radiation λ = 0.71073 Å
Hall symbol: P 2ac 2ab	Cell parameters from 9869 reflections
*a* = 8.3601 (2) Å	θ = 2.2–30.5º
*b* = 10.3596 (3) Å	µ = 0.31 mm^−^^1^
*c* = 20.5754 (6) Å	*T* = 100 (2) K
*V* = 1781.98 (8) Å^3^	Prism, colorless
*Z* = 4	0.43 × 0.34 × 0.25 mm
*F*_000_ = 784	

### Data collection

**Table:**

Bruker APEXII CCD area-detector diffractometer	6479 independent reflections
Radiation source: fine-focus sealed tube	5952 reflections with *I* > 2σ(*I*)
Monochromator: graphite	*R*_int_ = 0.042
*T* = 100(2) K	θ_max_ = 32.6º
φ and ω scans	θ_min_ = 2.0º
Absorption correction: multi-scan(APEX2; Bruker, 2005)	*h* = −12→12
*T*_min_ = 0.878, *T*_max_ = 0.927	*k* = −15→15
36558 measured reflections	*l* = −31→31

### Refinement

**Table:**

Refinement on *F*^2^	Hydrogen site location: inferred from neighbouring sites
Least-squares matrix: full	H-atom parameters constrained
*R*[*F*^2^ > 2σ(*F*^2^)] = 0.031	*w* = 1/[σ^2^(*F*_o_^2^) + (0.04*P*)^2^ + 0.35*P*] where *P* = (*F*_o_^2^ + 2*F*_c_^2^)/3
*wR*(*F*^2^) = 0.076	(Δ/σ)_max_ = 0.001
*S* = 1.01	Δρ_max_ = 0.39 e Å^−^^3^
6479 reflections	Δρ_min_ = −0.24 e Å^−^^3^
228 parameters	Extinction correction: none
Primary atom site location: structure-invariant direct methods	Absolute structure: Flack (1983), 2828 Friedel pairs
Secondary atom site location: difference Fourier map	Flack parameter: −0.01 (4)

### Special details

**Table:**

Geometry. All e.s.d.'s (except the e.s.d. in the dihedral angle between two l.s. planes) are estimated using the full covariance matrix. The cell e.s.d.'s are taken into account individually in the estimation of e.s.d.'s in distances, angles and torsion angles; correlations between e.s.d.'s in cell parameters are only used when they are defined by crystal symmetry. An approximate (isotropic) treatment of cell e.s.d.'s is used for estimating e.s.d.'s involving l.s. planes.
Refinement. Refinement of *F*^2^ against ALL reflections. The weighted *R*-factor *wR* and goodness of fit *S* are based on *F*^2^, conventional *R*-factors *R* are based on *F*, with *F* set to zero for negative *F*^2^. The threshold expression of *F*^2^ > σ(*F*^2^) is used only for calculating *R*-factors(gt) *etc*. and is not relevant to the choice of reflections for refinement. *R*-factors based on *F*^2^ are statistically about twice as large as those based on *F*, and *R*- factors based on ALL data will be even larger.

### Fractional atomic coordinates and isotropic or equivalent isotropic displacement parameters (Å^2^)

**Table:**

	*x*	*y*	*z*	*U*_iso_*/*U*_eq_	
S1	0.22775 (4)	0.63689 (3)	0.139002 (14)	0.01803 (7)	
S2	0.13141 (4)	0.84992 (3)	0.054416 (14)	0.01782 (7)	
N1	0.19500 (13)	0.60797 (10)	0.01133 (5)	0.01500 (19)	
N2	0.15503 (13)	0.65520 (9)	−0.05106 (5)	0.01352 (18)	
N3	0.25257 (12)	0.79575 (9)	−0.13046 (5)	0.01259 (18)	
N4	0.32207 (12)	1.01589 (9)	−0.10384 (5)	0.01291 (18)	
N5	0.32568 (13)	0.93935 (9)	−0.20956 (5)	0.01266 (18)	
C1	0.18653 (15)	0.68568 (11)	0.05930 (6)	0.0150 (2)	
C2	0.20870 (15)	0.77788 (11)	−0.06931 (5)	0.0123 (2)	
C3	0.29962 (14)	0.91713 (11)	−0.14572 (5)	0.01168 (19)	
C4	0.28199 (15)	0.99345 (11)	−0.04183 (5)	0.0132 (2)	
C5	0.21638 (15)	0.87583 (11)	−0.02250 (5)	0.0133 (2)	
C6	0.24582 (16)	0.46325 (12)	0.13017 (6)	0.0178 (2)	
H6A	0.2309	0.4225	0.1733	0.021*	
H6B	0.1592	0.4320	0.1014	0.021*	
C7	0.40534 (17)	0.42034 (15)	0.10245 (7)	0.0253 (3)	
H7A	0.4078	0.3259	0.0995	0.038*	
H7B	0.4919	0.4499	0.1309	0.038*	
H7C	0.4194	0.4574	0.0590	0.038*	
C8	0.13997 (14)	0.55459 (11)	−0.09830 (5)	0.0126 (2)	
C9	0.04771 (15)	0.57732 (12)	−0.15373 (6)	0.0151 (2)	
H9A	−0.0024	0.6587	−0.1599	0.018*	
C10	0.02966 (15)	0.48028 (13)	−0.19981 (6)	0.0170 (2)	
H10A	−0.0308	0.4963	−0.2381	0.020*	
C11	0.09974 (15)	0.35941 (13)	−0.19027 (6)	0.0178 (2)	
H11A	0.0866	0.2932	−0.2217	0.021*	
C12	0.18862 (15)	0.33680 (11)	−0.13451 (6)	0.0167 (2)	
H12A	0.2351	0.2543	−0.1276	0.020*	
C13	0.21026 (15)	0.43426 (11)	−0.08857 (6)	0.0151 (2)	
H13A	0.2727	0.4186	−0.0508	0.018*	
C14	0.39636 (15)	1.05860 (11)	−0.23480 (6)	0.0137 (2)	
H14A	0.4645	1.1009	−0.2017	0.016*	
H14B	0.3126	1.1199	−0.2491	0.016*	
C15	0.49585 (15)	1.01128 (13)	−0.29226 (6)	0.0163 (2)	
H15A	0.6028	0.9815	−0.2780	0.020*	
H15B	0.5087	1.0797	−0.3254	0.020*	
C16	0.39505 (16)	0.89890 (12)	−0.31828 (6)	0.0163 (2)	
H16A	0.3043	0.9306	−0.3447	0.020*	
H16B	0.4607	0.8394	−0.3449	0.020*	
C17	0.33637 (16)	0.83293 (11)	−0.25639 (6)	0.0151 (2)	
H17A	0.2306	0.7921	−0.2632	0.018*	
H17B	0.4132	0.7665	−0.2416	0.018*	
C18	0.30455 (17)	1.10308 (12)	0.00497 (6)	0.0187 (2)	
H18A	0.3845	1.1632	−0.0123	0.028*	
H18B	0.3412	1.0693	0.0469	0.028*	
H18C	0.2027	1.1484	0.0109	0.028*	

### Atomic displacement parameters (Å^2^)

**Table:**

	*U*^11^	*U*^22^	*U*^33^	*U*^12^	*U*^13^	*U*^23^
S1	0.02582 (15)	0.01779 (13)	0.01048 (11)	−0.00161 (12)	0.00034 (11)	0.00230 (10)
S2	0.02715 (16)	0.01428 (12)	0.01203 (12)	0.00202 (12)	0.00653 (11)	0.00012 (10)
N1	0.0204 (5)	0.0137 (4)	0.0109 (4)	−0.0006 (4)	0.0009 (4)	0.0031 (3)
N2	0.0203 (5)	0.0105 (4)	0.0098 (4)	−0.0011 (4)	0.0010 (3)	0.0008 (3)
N3	0.0161 (5)	0.0108 (4)	0.0109 (4)	0.0005 (3)	0.0010 (3)	0.0011 (3)
N4	0.0158 (5)	0.0110 (4)	0.0119 (4)	0.0002 (4)	0.0005 (3)	0.0002 (3)
N5	0.0181 (5)	0.0095 (4)	0.0104 (4)	0.0000 (3)	0.0019 (3)	0.0002 (3)
C1	0.0188 (5)	0.0144 (5)	0.0117 (5)	−0.0013 (4)	0.0020 (4)	0.0030 (4)
C2	0.0139 (5)	0.0110 (4)	0.0119 (5)	0.0008 (4)	0.0017 (4)	0.0005 (4)
C3	0.0125 (5)	0.0118 (4)	0.0108 (4)	0.0018 (4)	0.0006 (4)	0.0012 (4)
C4	0.0162 (5)	0.0114 (4)	0.0120 (4)	0.0012 (4)	−0.0002 (4)	−0.0005 (4)
C5	0.0176 (5)	0.0121 (5)	0.0102 (4)	0.0014 (4)	0.0021 (4)	0.0009 (4)
C6	0.0181 (6)	0.0169 (5)	0.0183 (5)	−0.0004 (4)	0.0000 (4)	0.0056 (4)
C7	0.0201 (6)	0.0296 (7)	0.0261 (7)	0.0060 (6)	−0.0007 (5)	0.0019 (5)
C8	0.0144 (5)	0.0109 (4)	0.0124 (4)	−0.0020 (4)	0.0023 (4)	−0.0006 (4)
C9	0.0160 (5)	0.0135 (5)	0.0158 (5)	−0.0003 (4)	0.0001 (4)	0.0014 (4)
C10	0.0160 (6)	0.0189 (6)	0.0162 (5)	−0.0023 (4)	−0.0012 (4)	−0.0011 (4)
C11	0.0185 (6)	0.0160 (5)	0.0189 (5)	−0.0034 (5)	0.0021 (4)	−0.0051 (4)
C12	0.0188 (5)	0.0118 (5)	0.0193 (5)	−0.0008 (4)	0.0035 (4)	−0.0003 (4)
C13	0.0170 (6)	0.0131 (5)	0.0152 (5)	0.0000 (4)	0.0013 (4)	0.0014 (4)
C14	0.0161 (5)	0.0117 (5)	0.0133 (5)	−0.0009 (4)	0.0013 (4)	0.0024 (4)
C15	0.0165 (5)	0.0202 (6)	0.0122 (5)	−0.0021 (4)	0.0011 (4)	0.0003 (4)
C16	0.0199 (6)	0.0187 (5)	0.0104 (5)	−0.0015 (5)	0.0010 (4)	0.0006 (4)
C17	0.0216 (6)	0.0115 (5)	0.0123 (5)	0.0006 (4)	0.0019 (4)	−0.0009 (4)
C18	0.0282 (7)	0.0136 (5)	0.0144 (5)	−0.0019 (5)	−0.0004 (5)	−0.0031 (4)

### Geometric parameters (Å, °)

**Table:**

S1—C1	1.7502 (12)	C8—C9	1.3968 (16)
S1—C6	1.8144 (13)	C9—C10	1.3901 (17)
S2—C5	1.7553 (11)	C9—H9A	0.9500
S2—C1	1.7657 (12)	C10—C11	1.3963 (19)
N1—C1	1.2757 (15)	C10—H10A	0.9500
N1—N2	1.4137 (13)	C11—C12	1.3868 (18)
N2—C2	1.3991 (14)	C11—H11A	0.9500
N2—C8	1.4307 (15)	C12—C13	1.3948 (16)
N3—C2	1.3235 (14)	C12—H12A	0.9500
N3—C3	1.3545 (14)	C13—H13A	0.9500
N4—C4	1.3394 (14)	C14—C15	1.5264 (17)
N4—C3	1.3508 (14)	C14—H14A	0.9900
N5—C3	1.3512 (14)	C14—H14B	0.9900
N5—C14	1.4646 (15)	C15—C16	1.5337 (18)
N5—C17	1.4670 (15)	C15—H15A	0.9900
C2—C5	1.4005 (15)	C15—H15B	0.9900
C4—C5	1.3942 (15)	C16—C17	1.5261 (16)
C4—C18	1.5008 (16)	C16—H16A	0.9900
C6—C7	1.5171 (19)	C16—H16B	0.9900
C6—H6A	0.9900	C17—H17A	0.9900
C6—H6B	0.9900	C17—H17B	0.9900
C7—H7A	0.9800	C18—H18A	0.9800
C7—H7B	0.9800	C18—H18B	0.9800
C7—H7C	0.9800	C18—H18C	0.9800
C8—C13	1.3926 (16)		
			
C1—S1—C6	102.06 (6)	C8—C9—H9A	120.2
C5—S2—C1	95.34 (5)	C9—C10—C11	120.48 (12)
C1—N1—N2	118.10 (10)	C9—C10—H10A	119.8
C2—N2—N1	118.84 (9)	C11—C10—H10A	119.8
C2—N2—C8	120.50 (9)	C12—C11—C10	119.50 (11)
N1—N2—C8	112.67 (9)	C12—C11—H11A	120.2
C2—N3—C3	115.51 (10)	C10—C11—H11A	120.2
C4—N4—C3	116.20 (10)	C11—C12—C13	120.52 (11)
C3—N5—C14	123.61 (10)	C11—C12—H12A	119.7
C3—N5—C17	121.36 (9)	C13—C12—H12A	119.7
C14—N5—C17	112.11 (9)	C8—C13—C12	119.73 (11)
N1—C1—S1	122.13 (9)	C8—C13—H13A	120.1
N1—C1—S2	125.35 (9)	C12—C13—H13A	120.1
S1—C1—S2	112.52 (7)	N5—C14—C15	102.92 (9)
N3—C2—N2	118.10 (10)	N5—C14—H14A	111.2
N3—C2—C5	122.66 (10)	C15—C14—H14A	111.2
N2—C2—C5	119.23 (10)	N5—C14—H14B	111.2
N4—C3—N5	117.95 (10)	C15—C14—H14B	111.2
N4—C3—N3	126.56 (10)	H14A—C14—H14B	109.1
N5—C3—N3	115.49 (10)	C14—C15—C16	102.40 (10)
N4—C4—C5	121.46 (10)	C14—C15—H15A	111.3
N4—C4—C18	116.64 (10)	C16—C15—H15A	111.3
C5—C4—C18	121.85 (10)	C14—C15—H15B	111.3
C4—C5—C2	117.07 (10)	C16—C15—H15B	111.3
C4—C5—S2	123.37 (8)	H15A—C15—H15B	109.2
C2—C5—S2	119.38 (9)	C17—C16—C15	103.02 (9)
C7—C6—S1	113.66 (10)	C17—C16—H16A	111.2
C7—C6—H6A	108.8	C15—C16—H16A	111.2
S1—C6—H6A	108.8	C17—C16—H16B	111.2
C7—C6—H6B	108.8	C15—C16—H16B	111.2
S1—C6—H6B	108.8	H16A—C16—H16B	109.1
H6A—C6—H6B	107.7	N5—C17—C16	103.36 (9)
C6—C7—H7A	109.5	N5—C17—H17A	111.1
C6—C7—H7B	109.5	C16—C17—H17A	111.1
H7A—C7—H7B	109.5	N5—C17—H17B	111.1
C6—C7—H7C	109.5	C16—C17—H17B	111.1
H7A—C7—H7C	109.5	H17A—C17—H17B	109.1
H7B—C7—H7C	109.5	C4—C18—H18A	109.5
C13—C8—C9	120.08 (11)	C4—C18—H18B	109.5
C13—C8—N2	121.16 (10)	H18A—C18—H18B	109.5
C9—C8—N2	118.72 (10)	C4—C18—H18C	109.5
C10—C9—C8	119.66 (11)	H18A—C18—H18C	109.5
C10—C9—H9A	120.2	H18B—C18—H18C	109.5
			
C1—N1—N2—C2	41.93 (16)	C18—C4—C5—S2	8.17 (17)
C1—N1—N2—C8	−168.98 (11)	N3—C2—C5—C4	−3.72 (18)
N2—N1—C1—S1	178.19 (9)	N2—C2—C5—C4	175.25 (11)
N2—N1—C1—S2	−1.02 (17)	N3—C2—C5—S2	171.64 (10)
C6—S1—C1—N1	−8.61 (13)	N2—C2—C5—S2	−9.38 (16)
C6—S1—C1—S2	170.70 (7)	C1—S2—C5—C4	−147.93 (11)
C5—S2—C1—N1	−33.47 (13)	C1—S2—C5—C2	37.01 (11)
C5—S2—C1—S1	147.25 (7)	C1—S1—C6—C7	78.51 (11)
C3—N3—C2—N2	178.42 (10)	C2—N2—C8—C13	127.99 (12)
C3—N3—C2—C5	−2.60 (18)	N1—N2—C8—C13	−20.53 (15)
N1—N2—C2—N3	143.21 (11)	C2—N2—C8—C9	−54.47 (15)
C8—N2—C2—N3	−3.42 (17)	N1—N2—C8—C9	157.01 (11)
N1—N2—C2—C5	−35.81 (16)	C13—C8—C9—C10	−1.54 (18)
C8—N2—C2—C5	177.56 (11)	N2—C8—C9—C10	−179.11 (11)
C4—N4—C3—N5	174.47 (11)	C8—C9—C10—C11	1.60 (19)
C4—N4—C3—N3	−5.39 (18)	C9—C10—C11—C12	−0.37 (19)
C14—N5—C3—N4	8.23 (17)	C10—C11—C12—C13	−0.94 (18)
C17—N5—C3—N4	167.32 (11)	C9—C8—C13—C12	0.25 (18)
C14—N5—C3—N3	−171.89 (10)	N2—C8—C13—C12	177.76 (11)
C17—N5—C3—N3	−12.81 (16)	C11—C12—C13—C8	1.00 (18)
C2—N3—C3—N4	7.60 (18)	C3—N5—C14—C15	144.67 (11)
C2—N3—C3—N5	−172.26 (10)	C17—N5—C14—C15	−16.12 (13)
C3—N4—C4—C5	−1.88 (17)	N5—C14—C15—C16	34.32 (12)
C3—N4—C4—C18	−179.27 (11)	C14—C15—C16—C17	−40.28 (12)
N4—C4—C5—C2	6.08 (18)	C3—N5—C17—C16	−170.29 (11)
C18—C4—C5—C2	−176.66 (12)	C14—N5—C17—C16	−9.01 (13)
N4—C4—C5—S2	−169.08 (9)	C15—C16—C17—N5	30.31 (13)

### Hydrogen-bond geometry (Å, °)

**Table:**

*D*—H···*A*	*D*—H	H···*A*	*D*···*A*	*D*—H···*A*
C12—H12A···N4^i^	0.95	2.62	3.5630 (15)	172
C15—H15B···S2^ii^	0.99	2.83	3.6264 (13)	138

Symmetry codes: (i) *x*, *y*−1, *z*; (ii) −*x*+1/2, −*y*+2, *z*−1/2.
